# Two Decades of Pediatric Inflammatory Bowel Disease in North-Western Romania: Phenotypic Characteristics and Diagnostic Trends

**DOI:** 10.3390/jcm14134597

**Published:** 2025-06-28

**Authors:** Georgia Valentina Tartamus (Tita), Daniela Elena Serban, Marcel Vasile Tantau

**Affiliations:** 1 3rd Medical Discipline, Department of Internal Medicine, “Iuliu Hatieganu” University of Medicine and Pharmacy, 400012 Cluj-Napoca, Romania; georgia.tartamus@umfcluj.ro; 2 Department of Mother and Child, 2nd Clinic of Pediatrics, Emergency Clinical Hospital for Children, “Iuliu Hatieganu” University of Medicine and Pharmacy, 400177 Cluj-Napoca, Romania; 3 Department of Internal Medicine and Gastroenterology, “Prof. Dr. Octavian Fodor” Regional Institute of Gastroenterology and Hepatology, “Iuliu Hatieganu” University of Medicine and Pharmacy, 400162 Cluj-Napoca, Romania; marcel.tantau@umfcluj.ro

**Keywords:** pediatric inflammatory bowel disease, Crohn’s disease, ulcerative colitis, IBD-unclassified, Paris classification, disease phenotype, diagnostic

## Abstract

**Background/Objectives**: Pediatric inflammatory bowel disease (pIBD), including Crohn’s disease (CD), ulcerative colitis (UC), and IBD-unclassified (IBD-U), exhibits unique clinical features compared to adult-onset disease. This study aimed to describe phenotypic characteristics of pIBD in the north-west region of Romania over a 21-year period and to compare our findings with those of other studies worldwide. **Methods**: We conducted a retrospective study of children under 18 years of age, from the north-west region of Romania, diagnosed with pIBD between 2000 and 2020 at the Emergency Clinical Hospital for Children, Cluj-Napoca. Disease phenotype at diagnosis was established according to the Paris classification. Data were collected from the hospital records and analyzed using descriptive statistics and univariate analysis of categorical variables. A *p*-value < 0.05 was considered statistically significant. **Results**: Ninety-four patients were included (CD: 51.0%; UC: 43.6%; IBD-U: 5.4%), with a median age at diagnosis of 14 years (11–15.7). Very early-onset IBD accounted for 5.3% of cases. The likelihood of being diagnosed with CD after 10 years of age was significantly higher compared to UC (OR = 4.75, 95% CI: 1.10–29.07, *p* = 0.03). UC most frequently presented as pancolitis (51.2%), while CD most often involved the ileocolonic region (56.3%). Inflammatory behavior was the most common CD phenotype (69%). Upper gastrointestinal involvement was documented in 18.7% of CD cases, with detection rates increasing after 2014. Perianal disease and growth impairment were significantly associated with complicated CD behavior (*p* = 0.03, and *p* = 0.007 respectively). Our findings are broadly consistent with other published reports. **Conclusions**: This study provides the first detailed phenotypic characterization of pIBD in this region. Our findings reflect trends observed in other populations and underscore the importance of standardized diagnostic evaluation.

## 1. Introduction

Inflammatory bowel disease (IBD) consists of Crohn’s disease (CD), ulcerative colitis (UC), and IBD-unclassified (IBD-U), all characterized by chronic, relapsing inflammation of the gastrointestinal tract, frequently accompanied by systemic symptoms and often associated with extraintestinal manifestations, affecting especially the skin, joints, eyes, or hepatobiliary system. While UC is limited to the colon and primarily affects the mucosal layer, CD can involve any part of the digestive tract and leads to transmural inflammation, increasing the risk of strictures and fistulas. IBD-U is diagnosed when features do not distinctly align with either CD or UC [1].

IBD is more common in adolescents and young adults, with nearly a quarter of patients experiencing their first symptoms before the age of 18 years [2,3]. Over the past three decades, a rising global incidence of pediatric IBD (pIBD) has been observed, particularly in newly industrialized countries and among younger children, signalling a possible shift in environmental and early-life risk factors [4,5].

The diagnosis of pIBD relies on a comprehensive assessment of clinical, laboratory, endoscopic, histologic, as well as radiologic findings, with the PORTO criteria of the European Society for Paediatric Gastroenterology, Hepatology, and Nutrition (ESPGHAN), revised in 2014, serving as the gold standard. These criteria emphasize the importance of performing upper and lower digestive endoscopies, and small bowel imaging to accurately distinguish between CD, UC, and IBD-U [1].

Several particular features characterize pIBD, including more extensive gastrointestinal involvement, a faster rate of early progression, and a higher likelihood of developing complicated disease phenotypes. Children with IBD are at an increased risk for growth impairment, delayed puberty, and reduced bone mineral density, which can have long-term consequences on their overall health. Additionally, the chronic nature of the disease, along with its treatment demands, often leads to significant psychosocial challenges, affecting emotional well-being, academic performance, and social interactions [6,7,8,9].

The Paris classification, an adaptation of the Montreal classification, was specifically developed to address the distinct features of pIBD. It introduces age-related categories to differentiate early-onset forms of the disease, classifying patients diagnosed before 10 years (A1a) separately from those diagnosed between 10 and 17 years (A1b). In CD, the classification refines disease location by incorporating upper gastrointestinal involvement (L4a: proximal to the ligament of Treitz, L4b: distal to the ligament of Treitz to above terminal ileum), while also distinguishing stricturing (B2), penetrating (B3), and a combination of both (B2B3) behaviors. In UC, it expands disease extension subtypes (E1-E4) and adds severity grading (S0: never severe, S1: ever severe). By accounting for the evolving nature of pIBD, the Paris classification enhances disease characterization, aiding in treatment decisions and long-term prognostic assessment [10].

Substantial variation in pIBD has been observed in terms of clinical presentation, disease phenotype, and outcomes across different populations, geographic regions, and ethnic groups. This variability is thought to result from a complex interplay of multiple factors, including age at diagnosis, sex, ethnicity, environmental exposures, geographic region of origin, and underlying genetic predisposition [11,12]. Furthermore, differences in diagnostic practices, access to specialized care, and patterns of disease recognition can also influence the reported distribution of phenotypes [13].

The aim of this study was to provide an analysis of the demographic characteristics and disease phenotypes of pIBD in the north-west region of Romania, covering a 21-year period. In addition to presenting regional data, this study compared our findings with those reported in other pIBD cohorts and registries worldwide. With this research, we intended to fill a significant gap in the literature, as most of the existing evidence on pIBD comes from North-Western Europe and North America, with limited data from Eastern Europe.

## 2. Materials and Method

### 2.1. Study Design

We performed a retrospective observational study analyzing pIBD cases diagnosed between 1 January 2000 and 31 December 2020 in the north-west region of Romania. This study was approved by the Ethics Committee of the ”Iuliu Hatieganu” University of Medicine and Pharmacy, Cluj-Napoca, Romania.

### 2.2. Patients

We included in the study cohort all consecutive pIBD patients under 18 years old of age, diagnosed at the Emergency Clinical Hospital for Children (ECHC) in Cluj-Napoca, who resided in one of the following six counties: Cluj, Maramureș, Satu Mare, Sălaj, Bistrița-Năsăud, and Bihor. The diagnosis of IBD was established based on the revised Porto criteria of the ESPGHAN [1]. Patients were categorized according to age at diagnosis as follows: adolescent-onset IBD (between 10 and 18 years old), early-onset IBD (between 6 and 9 years old), very-early-onset IBD (VEO-IBD, under 6 years old), and infantile onset IBD (under 2 years old) [10,14].

A previous study using this cohort focused on the incidence and temporal trends of pIBD in the north-west region of Romania [15]. This current research investigated disease phenotypes at diagnosis, which were not addressed in the earlier publication.

### 2.3. Methods

Patient data were sourced from both medical records and the hospital’s electronic database. The collected information included sex, age at diagnosis, year of diagnosis, IBD subtype (CD, UC, IBD-U), as well as disease phenotype.

Disease phenotype was attributed using the Paris classification [10]. The presence or absence of upper gastrointestinal endoscopy and small bowel imaging during the initial diagnostic evaluation was documented systematically for pIBD cases diagnosed from 2014 onward.

For UC, disease extension was classified as E1 for ulcerative proctitis, E2 for left-sided UC (distal to splenic flexure), E3 for extensive UC (distal to hepatic flexure), and E4 for pancolitis. Disease severity was assessed at diagnosis using the Pediatric Ulcerative Colitis Activity Index (PUCAI), with S0 indicating non-severe disease and S1 severe disease [10,16].

When considering CD cases, they were assigned to age categories at diagnosis as follows: A1a for patients younger than 10 years, A1b for those aged 10 to 16 years, and A2 for those older than 17 years. Disease location was categorized as L1, which involves the distal third of the ileum and/or limited cecal disease, L2, indicating colonic involvement, L3, representing ileocolonic disease, L4a and L4b, which refer to esophagogastric involvement, proximal and distal to the ligament of Treitz until terminal ileum, respectively. Disease behavior was classified as B1 for non-stricturing, non-penetrating disease, B2 for stricturing disease, B3 for penetrating disease, and B2B3 for both stricturing and penetrating disease. Perianal disease (p) was considered in the presence of perianal abscesses or fistulas, excluding isolated skin tags, fissures, or hemorrhoids. Growth impairment was evaluated using height-for-age z-scores and was recorded under the G category, with G0 indicating normal growth and G1 representing growth failure (Z score lower than minus 2 standard deviations) [10].

Cases with incomplete data on disease location, disease behavior, growth impairment status, or perianal disease presence (for CD), and on disease extension or severity (for UC), were excluded from analyses related to those specific variables.

We conducted a literature search of PubMed (National Library of Medicine, Bethesda, MD, USA) and Web of Science (Clarivate Analytics, Philadelphia, PA, USA) databases from inception through April 2025 to identify regional, national, or international studies assessing disease phenotypes for pediatric UC and pediatric CD. We analyzed the findings from the literature search in comparison to our study results and presented them in tables.

### 2.4. Statistical Analysis

Quantitative data were summarized as mean and standard deviation (SD) for normally distributed variables, and as median and interquartile range (IQR) for non-normally distributed variables. Qualitative variables were presented as counts and relative frequencies (%). For subgroup analyses, age at diagnosis was categorized into four groups: under 6 years, 6–9 years, 10–13 years, and 14–17 years. Associations between categorical variables were assessed using Chi-square or Fisher’s exact tests, with odds ratios (OR) and 95% confidence intervals (95% CI) calculated from contingency tables. All analyses were conducted for the entire dataset, as well as stratified by time of diagnosis—before 2014 (pre-2014) and from 2014 onward (post-2014). A *p*-value < 0.05 was considered statistically significant. Statistical analyses were performed using Microsoft Excel (Version 16.59) (Microsoft Corporation, Redmond, WA, USA) and R-Commander (Version 4.4.2) (R Foundation for Statistical Computing, Vienna, Austria).

## 3. Results

Ninety-four patients under 18 years of age, living in the north-west region of Romania were diagnosed with pIBD during the study timeframe. In our cohort, all pIBD subtypes were represented, and data on disease extension and behavior were available for the majority of cases, with missing complete information in 4.1% of CD and 17% of UC patients. Further details regarding patient demographics and IBD subtypes are presented in Table 1.

A higher proportion of males was observed in the CD group compared to the UC group; however, the difference was not statistically significant (*p* = 0.053).

In our cohort, the adolescent-onset IBD group comprised the largest proportion of patients, accounting for 84.1% (with 52.1% aged 14–17 years and 32% aged 10–13 years). Ten patients (10.6%) belonged to the early-onset IBD group, while five patients (5.3%) were in the VEO-IBD group. No cases were recorded in the infantile-onset IBD subgroup.

In the VEO-IBD group, 3 patients were diagnosed with UC (60%) and 2 with IBD-U (40%). All VEO-IBD patients with UC were female, while those with IBD-U were male. The 3 patients under 6 years of age with UC had varying disease extensions (E2, E3, and E4), with the E3 case showing a severe disease (E3S1).

Among patients with IBD under 10 years of age, UC was more common than CD (UC:CD ratio of 3.3:1). After 10 years of age, this trend reversed in favor of CD (UC:CD ratio of 1:1.45). The likelihood of being diagnosed with CD after 10 years of age was significantly higher compared to UC (OR = 4.75, 95% CI: 1.10–29.07, *p* = 0.03).

IBD-U was diagnosed in 2 patients under 10 years of age (specifically under 6 years), and in 3 patients over 10 years of age, representing 40% and 60% of the IBD-U cases, respectively.

At the initial diagnostic evaluation, upper gastrointestinal endoscopy was performed in 49.1% of cases, small bowel ultrasound in 78.6% (frequently using a water-enhanced technique), and magnetic resonance enterography in 14.7% of cases.

### 3.1. Ulcerative Colitis

**Figure 1:**
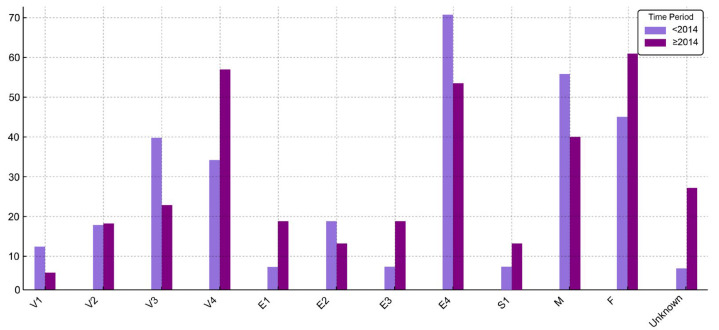
Distribution (%) of UC patient characteristics before 2014 and starting with 2014 (V1—age under 6 years; V2—age between 6 and 9 years; V3—age between 10 and 13 years; V4—age between 14 and 17 years).

Data on disease extension and severity were available for 34 patients (83%) diagnosed with UC; information was missing for one patient before 2014 and for 6 patients afterwards. Pancolitis (E4) was the most frequent presentation, observed in 51.2% of cases, followed by left-sided colitis (E2) in 12.2%. At diagnosis, severe disease (S1) was noted in 7.4% of patients. More information about disease extension is presented in Table 2. Additionally, a comparative overview of clinical and demographic characteristics of UC patients stratified by time of diagnosis (before 2014 and starting with 2014) is illustrated in Figure 1, highlighting shifts in disease extension, severity, age distribution, and sex over time.

No association was found between disease extension and either sex or age group in the overall cohort (*p* = 0.75 and *p* = 0.76, respectively). This lack of association remained consistent in time-stratified analyses.

A comparative analysis of disease phenotypes for UC cases across different pIBD studies is presented in Table 3. While most studies followed a prospective design, the Asian multicenter study employed a mixed approach, incorporating both retrospective and prospective data collection [17].

### 3.2. Crohn’s Disease

The majority of patients with CD belonged to the A1b age group (79.2%).

Information on disease location and behavior was available for 95.9% of patients with CD; data were missing for two cases, both diagnosed after 2014. Ileocolonic involvement (L3) was the most frequent location, observed in 56.3% of cases. Among male CD patients, L3 was the most common location (39.5%), while only a small proportion of females presented with L1 disease (2%); this distribution was consistent in both pre- and post-2014 analyses. We compared disease location by sex across the entire cohort, as well as within the subgroups diagnosed before 2014 and starting with 2014, but none reached statistical significance (*p* = 0.15, *p* = 0.7, and *p* = 0.1, respectively). Similarly, no significant association was observed between disease location and age group at diagnosis (*p* = 0.97). Esophagogastroduodenal involvement (L4a) was present in 18.7% of patients. One case was diagnosed before 2014 (6.7%) and 8 cases starting with 2014 (24.2%), but the difference was not statistically significant (*p* = 0.23). No cases of isolated L4 disease or L4b involvement were recorded. L4 disease was not associated with sex (*p* = 0.99), disease behavior (*p* = 0.59), perianal disease (*p* = 0.97), or growth impairment (*p* = 0.37). These findings remained consistent across time-stratified analyses, with no significant associations observed either before or starting with 2014.

The most common disease behavior was inflammatory (B1), observed in 69% of cases. Among those with B1 behavior, the male-to-female ratio was 2.6:1, whereas for other behavior types, the ratio was approximately 1:1. When analyzed by time of diagnosis, B1 remained the predominant phenotype, increasing from 60% before 2014 to 72.6% thereafter. In contrast, the proportion of patients with combined penetrating and stricturing behavior (B2B3) declined from 20% to 3.1% over the same period. These temporal changes did not reach statistical significance (*p* = 0.25). No significant differences were observed between disease behavior and sex or age group, both in the entire cohort and in time-stratified analyses. While no relationship between disease location and behavior was observed in patients diagnosed before 2014, a statistically significant association emerged in the post-2014 cohort (*p* = 0.01) and remained evident in the full dataset (*p* = 0.02), driven primarily by a predominance of ileocolonic involvement (L3) among those with B1 behavior.

The frequency of perianal disease in our cohort was 10.4%. It was more common among patients with complicated disease behavior (B2, B3, B2B3) compared to those with inflammatory behavior (B1). The association was statistically significant in the overall dataset and in time stratified analysis (*p* = 0.03, *p* = 0.001, and *p* = 0.008 respectively). However, perianal disease was not associated with disease location (separated for L4), sex, or age group in either the overall cohort or time-stratified analyses.

Growth impairment was observed in 33.3% of CD patients, occurring more frequently in those with L1 and L3 disease compared to isolated colonic involvement (L2). However, no significant relationship was identified between growth impairment and disease location (separated for L4), or with sex, and age group in any of the analyses. All patients with B2B3 behavior exhibited growth impairment, and it was also more common among those with B2 behavior than B1. Growth impairment was significantly associated with complicated disease behavior (B2, B2B3) in the overall cohort and in patients diagnosed before 2014 (*p* = 0.007, and *p* = 0.03 respectively). However, this association was not statistically confirmed in the post-2014 group, despite a similar trend (*p* = 0.1).

**Figure 2:**
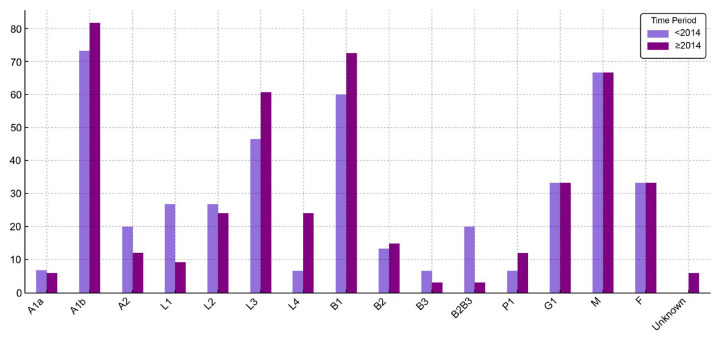
Distribution (%) of CD patient characteristics before 2014 and starting with 2014. (P1—present perianal disease).

Further details regarding the classification of CD cases according to the Paris classification are presented in Table 4. Also, a comparative visualization of clinical and demographic characteristics of CD patients, stratified by time of diagnosis (before 2014 and starting with 2014), is presented in Figure 2.

Table 5 provides a cross-study comparison of CD disease phenotypes in pIBD cohorts. Most studies employed a prospective design; however, the Asian multicenter study combined retrospective and prospective data collection, while the Korean study was conducted retrospectively [17,22].

## 4. Discussion

This 21-year retrospective study of pIBD in the north-west region of Romania identified 94 cases, with a predominance of CD (51.0%), followed by UC (43.6%) and IBD-U (5.4%), the majority presenting during adolescence. A small but clinically important subgroup was diagnosed with VEO-IBD. Among children under 10 years old, UC was more frequent than CD, but after 10 years of age, this trend reversed in favor of CD. Ileocolonic involvement and an inflammatory (B1) phenotype were most frequently observed in CD cases, whereas UC typically presented as extensive colitis (E3/E4). The majority of cases with upper GI involvement (L4) were identified after 2014.

When analyzing the demographic sex characteristic in our study cohort, males and females were almost equally distributed among all pIBD cases. We observed a male predominance in the CD group compared to the UC group, the difference being close to statistical significance. Studies in Europe have reported similar trends, with a higher rate of CD diagnoses in boys compared to girls during childhood and adolescence [18,19].

The median age at diagnosis in our cohort was 14 years, closely aligning with reports from other regions, including the French EPIMAD registry (14.3 years) [19] and a Canadian cohort (13 years) [27], supporting the observation that pIBD predominantly presents in children over 10 years old. Notably, a younger median age was reported from the Italian registry (11.7 years) [2].

VEO-IBD is recognized as a subtype of pBD with distinct clinical characteristics. In our cohort, VEO-IBD accounted for 5.3% of all cases, consistent with findings from the Italian Registry (4%), French registry (3%), and Israeli national cohort (4.8%) [2,14,28]. In contrast, a multicenter Asian pediatric IBD registry reported a substantially higher frequency of VEO-IBD (25.5%), highlighting a notable regional variability [29]. Data from Canada, where pIBD incidence is among the highest globally, showed an increase in the incidence of VEO-IBD between 1999 and 2010 [27]. Such differences suggest that both environmental exposure and genetic susceptibility may influence the occurrence of VEO-IBD. Disease in this age group tends to present with colonic involvement more frequently than in older children or adults [30], a trend confirmed in our study where all VEO-IBD patients were diagnosed with either UC or IBD-U, with no cases of CD reported. Children with onset of disease at younger ages have lower rates of hospitalisation, emergency department utilization and surgical resection [31]. However, infantile-onset IBD has been sometimes associated with poorer long-term outcomes, as noted in the Israeli cohort [14]. Given the prolonged disease course expected in the VEO-IBD population, further longitudinal studies are required to better understand long-term outcomes.

The proportion of newly diagnosed pIBD patients with CD increases with age at presentation. The predominance of CD over UC observed in our cohort, both overall and in the >10-years age group, is consistent with findings from other pediatric studies [18,19]. A lower prevalence of CD over UC was reported in the Italian pIBD population [2]. These findings also stand in contrast to adult epidemiological data [32].

UC in pediatric patients has a high tendency toward extensive disease, as previously stated [7]. Our data are consistent with these observations, with pancolitis (E4) detected in 51.2% of cases and the extensive form (E3) in 9.8%, totaling 70%. Similar proportions were reported in the Italian registry (E3 + E4: 64%) [2]. When examining UC characteristics over time, certain differences emerge. From 2014 onward, a higher proportion of patients were aged over 14 years old, and limited disease extension (E1) was more frequently observed. However, the overall distribution of adolescent patients remained relatively stable across both time periods (72.1% pre-2014, and 78.2% post-2014). Similarly, the proportion of patients with extensive disease involvement (E3 and E4 combined) showed minimal variation, decreasing only slightly from 76.5% before 2014 to 70.5% thereafter. Overall, the data suggest minor temporal shifts, while the core clinical profile of UC remained largely consistent. Our study did not follow patients longitudinally; therefore, disease severity was assessed only at presentation and was observed in 7.3% of cases. A slightly higher proportion of patients were reported with severe disease at diagnosis in the Hungarian registry (18.6%) [18].

Extensive disease at diagnosis is clinically relevant, as it may predict the need for advanced therapies (e.g., anti-tumor necrosing factor), and influence treatment response. In contrast, localized forms such as proctitis (E1) may initially be managed with topical treatments (e.g., mesalazine), which can be effective while minimizing systemic exposure. However, limited disease may still progress over time or respond poorly to local therapy, ultimately requiring escalation to systemic treatment [33,34,35]. In pediatric UC, greater disease extent and a high PUCAI score (≥65) at presentation have been associated with an increased risk of colectomy. While disease extent can inform initial therapeutic decisions, it does not appear to be a reliable predictor of relapse [36]. Data from the HUPIR registry reported no variation in disease location based on age at diagnosis or sex [18]. Our findings were consistent with these observations; however, the interpretation of age-related associations should be made with caution, as small sample sizes—particularly in the youngest age subgroups—may have limited the statistical power to detect meaningful differences.

When analyzing CD patients by age at diagnosis, the majority were diagnosed between 10 and 17 years, accounting for over three-quarters of cases, consistent with findings from other pIBD cohorts [17,18,24]. The proportion of children diagnosed before the age of 10 was 6.2% in our study, which is relatively close to that reported in the Hungarian HUPIR registry (10.9%) [18], but substantially lower than in the Italian registry (23.5%) [2] and the Czech cohort (27.6%) [20]. This discrepancy may highlight the need to increase awareness among Romanian pediatricians regarding the possibility of early-onset and very early-onset IBD, which, although less common, should not be overlooked.

The distribution of disease location in our cohort is comparable to prior reports, with ileocolonic disease (L3) being the most common phenotype, accounting for approximately half of cases, followed by isolated colonic (L2) and ileal (± cecal) disease (L1) [2,7,18,23,25]. Between L1 and L2 phenotypes, L2 is generally reported as more frequent, though findings vary [17,21,23]. The observation that colonic involvement is common in pediatric CD [7] is also reflected in our data, where the combined prevalence of L2 and L3 exceeded that of L1 and L3. While previous studies have suggested that isolated colonic disease is more frequent in early- and very-early-onset CD, with ileal involvement increasing with age [2,7,23], our findings did not confirm this trend, as no significant association was observed between disease location and age groups at diagnosis. However, this result should be interpreted with caution, as the number of patients in younger age subgroups was small (3 CD patients under 10 years), limiting the statistical power to detect age-related differences. Likewise, differences in disease location between male and female patients were not significant, consistent with findings from the EUROKIDS registry [23].

Disease location in pediatric CD has important prognostic implications. Genetic factors, such as NOD2/CARD15 variants, have been shown to be predictive of ileal involvement [37], reinforcing the role of genetic predisposition in disease localization. Isolated colonic disease (L2) is generally associated with a lower risk of surgery, whereas involvement of the small bowel (L1 and L3), with or without proximal extension (L4b), has been linked to a higher likelihood of developing stricturing (B2) and potentially penetrating (B3) complications [37,38]. In the overall analysis from our cohort, a significant association was found between disease location and behavior, characterized by a predominance of ileocolonic involvement (L3) among patients with inflammatory behavior (B1). However, when stratified by time of diagnosis, this association was observed only in the post-2014 cohort. This finding may, at least in part, reflect advancements in diagnostic practices. The routine implementation of full colonoscopy with ileoscopy, along with increased use of cross-sectional imaging modalities, likely enhanced the detection of ileal and transmural disease, contributing to more accurate phenotype classification in recent years.

Disease location at diagnosis not only informs prognosis but also guides the selection of initial therapy in pediatric CD. Budesonide is typically reserved for mild-to-moderate ileal disease (L1) due to its targeted release in the terminal ileum and right colon, thereby reducing systemic corticosteroid exposure [39]. In cases of isolated colonic involvement (L2), some studies have suggested reduced efficacy of exclusive enteral nutrition (EEN) for induction of remission [40]; however, current treatment guidelines recommend EEN as first line therapy in active luminal CD irrespective of disease location [39]. Mesalazine may occasionally be considered in very mild L2 disease [38], given its effectiveness in UC, though its utility in pediatric CD remains limited and is not routinely endorsed [41].

An important feature of pediatric CD is the frequent involvement of the upper gastrointestinal (GI) tract (30–70%) [18,42], which is significantly higher compared to adult-onset CD, where it is reported in 6.5–16.4% of cases [43,44,45]. Notably, upper endoscopy is recommended in adult CD patients only in the presence of upper GI symptoms [46]. In the Italian registry, isolated L4 disease was observed in 4.4% of cases and L4 association in 40.8% [2], while the Hungarian registry reported a similar frequency of L4 association but a lower prevalence of isolated L4 (0.4%) [18]. In contrast, a Korean study reported a significantly higher prevalence of upper GI involvement (74.4%), likely due to the systematic use of upper endoscopy at diagnosis and exclusion of patients without complete gastrointestinal evaluation [47]. The higher rate of L4 involvement in the Korean cohort, along with ethnic differences noted in the EUROKIDS registry, where Caucasian patients were less likely to exhibit L4b disease than non-Caucasian patients (22% vs. 41%) [23], may suggest a genetic predisposition to upper GI disease among non-Caucasian populations.

In our cohort, a lower prevalence of upper GI involvement was observed (18.7%), exclusively in the form of L4a disease, with no cases of isolated L4 or L4b phenotypes. Only one case was identified before 2014, whereas eight additional cases were diagnosed afterward, but the difference was not statistically significant. This likely reflects the adoption of the 2014 revised Porto criteria of ESPGHAN, which emphasize the use of systematic upper endoscopy and small bowel imaging in the diagnostic evaluation of pIBD [1]. Among cases diagnosed after 2014, upper GI endoscopy was performed at diagnosis in 49.1% of patients, small bowel ultrasound (often using a water-enhanced technique) in 78.6%, and MRE in 14.7%. These data suggest that the increased detection of L4a disease after 2014 may reflect improved diagnostic practices, particularly the more frequent use of endoscopic and imaging techniques, alongside epidemiological trends such as the rising incidence of pediatric CD in the same region [15]. However, the inconsistent application of full upper GI evaluation may have contributed to the lower detection rate of L4a disease compared to other data and the absence of L4b and isolated L4 phenotypes in our cohort. This variability likely stems from a combination of healthcare system limitations and institution-specific practices. At the time, Romania was classified as a lower-middle-income country [48], which may have limited healthcare resources. Contributing factors likely included restricted access in our hospital to anesthesiologists required for deep sedation in our hospital, which is essential for complete procedures, and occasional reliance on adult gastroenterology units for endoscopic procedures—where upper GI endoscopy is not routinely performed for IBD diagnosis in the absence of symptoms. Similar discrepancies in diagnostic thoroughness have been reported in large registries such as EUROKIDS, where only 60% of patients underwent complete assessment, often lacking terminal ileum intubation or upper GI endoscopy.

Our findings also support previous observations that upper GI involvement is more commonly associated with ileocolonic disease. Seven patients (14.6%) presented with the maximum disease extent (L3 + L4) at diagnosis, a rate comparable to the Hungarian pIBD registry (19.8%) [18], but lower than that reported by Van Limbergen et al. (27%) [7]. Beyond its association with broader disease distribution [49], the L4 phenotype has been linked to a greater need for aggressive therapy [50], although its impact on long-term prognosis remains controversial [51]. Furthermore, emerging evidence suggests that the prognostic impact varies by location, with L4b disease associated with a higher risk of poor outcomes compared to L4a involvement [51].

Regarding disease behaviour, the most frequent pediatric CD type is inflammatory (B1), followed by stricturing (B2) and then penetrating (B3) phenotypes. Our cohort reflected this trend, with reported frequencies of 69% for B1, 14.6% for B2, and 4.1% for B3. When compared to other European studies, the prevalence of B1 was lower in our cohort (92% in a Scottish cohort, and 82% in the EUROKIDS registry), while the frequency of B2 was higher than in the Scottish cohort (4%), but similar to other studies (12% in the EUROKIDS registry) [7,20,23].

The difference in frequency of the B1 and B2 phenotypes may reflect difficulties in precisely distinguishing inflammatory stenotic from fibrous stenotic disease. Although cross-sectional imaging techniques such as ultrasound (US), computed tomography enterography (CTE), and MRE are highly accurate in diagnosing CD—associated strictures, differentiating between fibrotic and inflammatory components remains a challenge [52,53,54]. Accurate differentiation is clinically important, as inflammatory lesions may respond to medical therapy, including corticosteroids or biologics, whereas fibrotic strictures typically require endoscopic dilation or surgical intervention due to their limited responsiveness to anti-inflammatory treatment [41,55].

A relatively high percentage of the complicated B2B3 phenotype was identified in our cohort (8.2%), whereas other European studies report considerably lower frequencies −2% in the EUROKIDS registry [23], and 0.5% in the Czech cohort [20]. Although time-related differences in disease behavior did not reach statistical significance, the observed increase in B1 and decline in B2B3 phenotypes starting with 2014 may reflect improvements in early detection and clinical management. Earlier diagnosis—potentially facilitated by the existence of pediatric gastroenterology specialists and improved access to specialized care and diagnostic modalities—could have contributed to identifying patients before progression to more complicated disease behavior at the time of classification.

According to EUROKIDS, inflammatory behavior (B1) is more commonly observed in children under 10 years of age, while complicated phenotypes (B2 and B2B3) tend to appear more frequently in patients diagnosed after the age of 10 years [23]. Evidence from larger studies suggests that older age at CD onset may increase the risk of developing internal penetrating complications (B3), though not stricturing disease (B2) [37]. However, our findings did not reproduce this association, possibly due to the small number of patients under 10 years of age, which may have limited the power to detect age-related variations. The differences between genders regarding disease behaviour were not significant and these observations are similar to those from the EUROKIDS registry [23]. Stricturing and penetrating phenotypes in pediatric CD are indicators of more severe disease, often requiring surgical intervention and escalation of medical therapy [37]. The presence of B3 behavior typically warrants the use of biologic agents, such as anti–tumor necrosis factor medications [39]. Such treatment implies long-term immunosuppression, requires careful monitoring for adverse effects and loss of response, and may pose an economic burden on the healthcare system as well as psycho-social challenges for patients and families.

The observed perianal disease frequency (10.4% of CD cases) is comparable to rates reported in other pediatric studies [20,23]. In our cohort, perianal disease was more frequently observed among patients with complicated disease behavior (B2, B3, B2B3) compared to those with inflammatory behavior (B1); the association reached statistical significance in the overall dataset and in time stratified analysis. Previous studies have supported the notion that perianal involvement in pediatric CD can be correlated with an increased risk of future stricturing and internal penetrating complications. Older age at disease onset and ethnicity (Black and South Asian children and adolescents) have been associated with an increased likelihood of developing perianal involvement. Recent evidence also suggests that male sex may influence the development of perianal complications [37]. However, our data did not support an association between sex or age at diagnosis and the presence of perianal disease. It is worth noting that the small sample size, particularly in younger age categories, may have limited our ability to identify subtle or clinically relevant associations.

The percentage of growth delay at diagnosis in the CD cases from our study (33.3%) was at the higher end of the previously reported range (4% to 38%). This wide variation in reported growth failure at diagnosis may be due to differences in its definition, the type of study conducted, and regional variations in growth standards or reference values used for anthropometric assessment [56,57]. Growth impairment is recognized as a clinically significant marker in pediatric CD, as it has been associated with an increased risk of bowel surgery [37]. In our cohort, growth impairment was more frequently observed in patients with complicated disease behavior (B2, B2B3) and was significant in the overall cohort. The association between growth impairment and complicated disease behavior observed in the pre-2014 subgroup and absent in the post-2014 subgroup, together with the stable prevalence of growth delay across both time periods, is likely explained by the higher frequency of B2B3 behavior during the earlier period. As previously discussed, timelier diagnosis in the more recent period may have prevented progression to complicated phenotypes by the time of classification, reducing the apparent link between growth failure and disease behavior. Although isolated small bowel disease (L1) has been linked to a greater risk of linear growth impairment in the literature, our findings showed only a trend in that direction, without reaching statistical significance. Similarly, sex and age at diagnosis were not associated with growth impairment in our cohort, despite previous reports suggesting a higher risk in males and in those diagnosed at a younger age [37]. However, this finding should be interpreted with caution due to the limited sample size in younger age groups, which may have reduced the statistical power to detect meaningful associations.

Early identification and intervention are crucial, as persistent growth impairment can lead to long-term consequences, including reduced adult height and compromised bone health [58]. According to recent consensus statements, the presence of growth failure at diagnosis is a marker of high-risk disease, warranting early escalation to biologic therapy to improve both disease control and growth outcomes [37]. Therefore, minimizing corticosteroid exposure and prioritizing EEN or other steroid-sparing therapies can optimize growth and development in pIBD.

IBD-U typically refers to patients who present with UC-like features but also exhibit characteristics suggestive of CD. In our retrospective study, IBD-U accounted for 5.4% of cases, a proportion slightly lower than those reported in national registries from Hungary (6%), Spain (7.3%), and Italy (7.4%) [2,18,25]. This supports previous observations that IBD-U is more frequently reported in prospective studies than in retrospective ones [59]. In terms of demographics, our cohort showed a higher proportion of male patients (80%) and a younger median age at diagnosis (11 years), compared to the EUROKIDS registry (52% male, median age 12.3 years). Although they reported that IBD-U was more commonly diagnosed in children under 10 years of age, this trend was not observed in our cohort. Notably, the same registry demonstrated a decrease in IBD-U diagnoses from 7.7% at baseline to 5.6% after follow-up, emphasizing the importance of a thorough and repeated diagnostic workup. Our study did not have a follow-up component which may have hindered the reassignment of some IBD-U cases to CD or UC. Winter et al. further highlighted that IBD-U diagnosis is less likely when patients undergo a complete endoscopic evaluation and small bowel imaging [13].

To improve diagnostic precision in pIBD, with a focus on resource-limited settings, several strategies could be considered. First, institutional diagnostic protocols should be regularly updated to reflect current international standards, such as ECCO-ESPGHAN guidelines. Ensuring adherence to these protocols through periodic evaluation may help reduce variability in clinical practice. For teaching hospitals, investing in the education of pediatricians—especially pediatric gastroenterologists, but also radiologists, and adult gastroenterologists involved in pediatric care, can enhance familiarity with diagnostic algorithms and improve procedural skills. Establishing dedicated pIBD teams, including pediatric gastroenterologists, radiologists, pathologists, and dietitians, may support a more coordinated and accurate diagnostic approach. Additionally, providing patients and families with accessible and structured information about the diagnostic process can foster understanding and engagement. Beginning with 2020, age-appropriate educational material—namely, the *Professor Nimbal* comic book—was introduced in Romania with the support of the Romanian IBD Patient Association [60,61]. Collectively, these measures aim to support timely diagnosis, with further targeted treatment, and improved outcomes in children with IBD.

Our study has several limitations, primarily its retrospective design, which may introduce selection and information biases.

Being a single-centre study, findings may not be fully representative of broader populations. However, the ECHC in Cluj-Napoca comprises three pediatric departments and includes a dedicated pediatric gastroenterology unit. In the early 2000s, it functioned as a national tertiary referral center for pIBD, particularly following the specialized training completed in 1999 by one of the doctors and present author (D.E.S) at a renowned pediatric gastroenterology clinic abroad. There, expertise was gained in colonoscopy and the diagnostic and therapeutic management of pIBD. In subsequent years, another pIBD care service became available at a medical center in the capital city. Currently, five pIBD referral centers are recognized nationally, with the ECHC in Cluj-Napoca serving as the primary center for the North-West region. Consequently, retrospective comparative data from other Romanian centers—particularly for the period between 2000 and 2010—are not available. Moreover, the absence of a national pIBD registry in Romania has further limited access to comprehensive, population-level data.

In Romania, pediatric patients (<18 years) with IBD are generally managed within specialized pediatric care settings, as adult gastroenterologists are not typically authorized to treat pediatric cases. Additionally, prescription of IBD-related medication requires formal documentation from a pediatric specialist, and primary care providers do not initiate or manage treatment independently. Given the universal healthcare coverage for children in Romania and the limited involvement of private medical services, pIBD care is largely centralized within the public healthcare system. Therefore, we can confidently assume that the vast majority of regional pIBD cases were captured within this cohort.

The inconsistent application of small bowel imaging and upper gastrointestinal endoscopy, particularly before 2014, may have contributed to under-recognition of L4 involvement and misclassification of disease extent, impacting the Paris classification.

Additionally, the small sample size in certain subgroups—particularly among younger age groups—may have limited the statistical power to detect meaningful associations. This limitation is partly attributable to the low incidence of pIBD in the North-West region during the study period [15]. Consequently, results from these analyses should be interpreted with caution. Also, multivariate analysis was not performed due to the limited number of cases within certain subgroups, particularly younger age categories and patients with less common disease phenotypes. Including multiple covariates under these conditions would risk overfitting and unstable estimates. Therefore, the analyses were restricted to univariate methods to preserve statistical validity.

A further limitation of our study is the absence of full data, most notably regarding UC disease extension and severity (17%) and to a lesser extent for CD phenotype classification (4.1%). Due to the retrospective nature of the study, missing data likely resulted from incomplete documentation or diagnostic procedures. Based on available records, no systematic pattern of missingness was identified. Moreover, the lack of follow-up data limited our ability to evaluate long-term disease evolution. These limitations highlight the need for future prospective, multicenter studies incorporating standardized data collection and longitudinal follow-up, to better characterize pIBD in Romania and ultimately improve patient outcomes. The establishment of a national pIBD registry would be a key prerequisite for enabling such studies, providing a structured framework for comprehensive data gathering and long-term monitoring.

## 5. Conclusions

This 21-year retrospective study provides an overview of pIBD phenotypic characteristics in the north-west region of Romania and represents one of the few detailed analyses from Eastern Europe. The findings confirm that pIBD most frequently presents in adolescence and is characterized by a predominance of ileocolonic disease in CD and extensive colitis in UC. Although inflammatory behavior was the most common phenotype, a notable proportion of CD patients exhibited complicated disease behavior (stricturing and/or penetrating disease) that was significantly associated with perianal disease and growth impairment. A minority of patients represented a clinically distinct and relevant subgroup with VEO-IBD, characterized by predominantly colonic disease. Upper GI involvement was likely underrecognized due to incomplete diagnostic workups, particularly in earlier years. The retrospective nature of the study, reliance on historical records, and relatively small cohort size may limit the generalizability and robustness of the findings. Moreover, temporal changes in diagnostic protocols and healthcare access may have influenced case ascertainment and classification. Despite these limitations, our findings are broadly consistent with patterns reported in most published studies to date. Also, they highlight the importance of recognizing less common pIBD subtypes early, adhering to standardized diagnostic protocols, and ensuring accurate classification.

## Figures and Tables

**Table 1:** Characteristics at diagnosis of pediatric inflammatory bowel disease (pIBD) patients, diagnosed between 2000 and 2020 in the north-west region of Romania.

	pIBD		UC		CD		IBD-U	
**Number of patients (%)**	94 (100)		41 (43.6)		48 (51.0)		5 (5.4)	
**Sex**	F	M	F	M	F	M	F	M
**Number of patients** **(% of disease type)**	39 (41.5)	55 (58.5)	22 (53.7)	19 (46.3)	16 (33.4)	32 (66.6)	1 (20)	4 (80)
**Median Age (years)** **(IQR)**	14 (11–15.7)		13.1 (9.6–15)		14.5 (11.5–16.5)		11 (5.5–14.6)	
**Age** **(years)**	Max.	Min.	Max.	Min.	Max.	Min.	Max.	Min.
	17.9	3.5	17.9	3.5	17.5	6.8	17.1	4.7

UC—ulcerative colitis, CD—Crohn’s disease, IBD-U—inflammatory bowel disease—unclassified, F—female, M—male, IQR—interquartile range, Max.—maximum, Min.—minimum).

**Table 2:** Disease extension and severity according to the Paris classification for UC cases diagnosed between 2000 and 2020 in the north-west region of Romania.

	E1	E2	E3	E4	S0	S1	Unknown
**Number of Patients** **(%)**	4 (9.8)	5 (12.2)	4 (9.8)	21 (51.2)	31 (75.6)	3 (7.4)	7 (17.0)

E1—ulcerative proctitis; E2—distal to splenic flexure; E3—distal to hepatic flexure; E4 –proximal to hepatic flexure; S0—non-severe disease at diagnosis; S1—severe disease at diagnosis—severity defined by the Pediatric Ulcerative Colitis Activity Index ≥ 65) [10,16].

**Table 3:** Pediatric UC phenotype comparison between different studies (according to the Paris classification) [2,17,18,19,20,21].

	Phenotype	E1 (%)	E2 (%)	E3 (%)	E4 (%)	S0 (%)	S1 (%)
Study							
Romanian cohort (2000–2020) *		9.8	12.2	9.8	51.2	75.6	7.4
Italian Registry (2009–2018)		6.6	29.4	9.2	54.8	92.7	7.3
Hungarian Registry (2007–2009)		5	24.8	13.2	57	81.4	18.6
French Registry (1988–2011)		27.8	29.5	10.5	32.2	-	-
Czech cohort (2002–2017)		4.9	13	6.5	73.2	-	-
Multi-centre Asian Registry (1995–2019) *		7.5	15.1	4.7	72.6	78.2	21.8
Japanese Registry (2012–2015)		6.8	12.3	4.8	76	78.5	21.5

(* retrospective studies).

**Table 4:** Disease classification according to the Paris classification for CD cases diagnosed between 2000 and 2020 in the north-west region of Romania.

	Age at Diagnosis			Location						
	A1a	A1b	A2	L1	L2	L3	L4 (a and/or b)	L1 + L4	L2 + L4	L3 + L4
**Number of patients ** **(%)**	3 (6.2)	38 (79.2)	7 (14.6)	7 (14.6)	12 (25.0)	27 (56.3)	9 (18.7)	0	2 (4.1)	7 (14.6)
	**Growth**		**Behavior**				**Perianal Disease**		**Unknown**	
	**G0**	**G1**	**B1**	**B2**	**B3**	**B2B3**	**Present**	**Absent**		
**Number of patients** **(%)**	30 (62.6)	16 (33.3)	33 (69)	7 (14.6)	2 (4.1)	4 (8.2)	5 (10.4)	41 (85.5)	2 (4.1)	

(A1a: < 10 years, A1b: 10−16 years, A2: 17–40 years; L1: distal 1/3 ileum/limited cecal disease, L2: colonic, L3: ileocolonic, L4—upper gastrointestinal disease associated with other locations; L4a: upper disease proximal to ligament of Treitz, L4b: upper disease, distal to ligament of Treitz and proximal to distal 1/3 ileum; G0: no evidence of growth delay, G1: growth delay; B1: nonstricturing nonpenetrating, B2: stricturing, B3: penetrating; B2B3: both penetrating and stricturing disease) [10].

**Table 5:** Pediatric CD phenotype comparison between different studies (according to the Paris classification) [2,17,18,19,20,21,22,23,24,25,26].

	Phenotype	A1a (%)	A1b (%)	A2 (%)	L1 (%)	L2 (%)	L3 (%)	L4 (isolated) (%)	L4a (%)	L4b (%)	B1 (%)	B2 (%)	B3 (%)	B2B3 (%)	Perianal Disease (%)	Growth Impairment (G1) (%)
Study																
Romanian cohort (2000–2020) *		6	79	14	14	25	56	0	18	0	69	14	4	8	10	33
Italian Registry (2009–2018)		-	-	-	25	27	41	4	40		-	-	-	-	13	-
Hungarian Registry (2007–2009)		10	74	9	13	27	58	0.4	30	13	84	12	2	1	14	6
EUROKIDS Registry (2004–2009)		-	-	-	16	28	53	4	30	24	82	12	5	2	9	-
French Registry (1988–2011) ^		-	-	-	12	15	71	-	21		77	18	4	-	6	-
Czech cohort (2002–2017)		27	59	12	-	-	69	-	14		75	12	8	0.5	11	29
New Zealand cohort—under 16 years old (1996–2015) ^		25	74	-	18	21	56	0.7	39	4	81	11	3	3	19	-
Korean Single Centre (1987–2013) *		2	73	23	10	8	79	1	6	17	88	9	1	0.1	47	11
Multi-centre Asian Registry (1995–2019) *		-	-	-	18	36	41	4	40	8	90	3	5	0.7	13–30	18
Japanese Registry (2012–2015)		21	79	0	18	13	64	2	47	20	83	11	3	2	34	7

* retrospective studies; ^ : The authors calculated percentages using information from the study).

## Data Availability

The original contributions presented in this study are included in the article; further inquiries can be directed to the corresponding author, within the legal regulations.
